# Quantifying critical states of complex diseases using single-sample dynamic network biomarkers

**DOI:** 10.1371/journal.pcbi.1005633

**Published:** 2017-07-05

**Authors:** Xiaoping Liu, Xiao Chang, Rui Liu, Xiangtian Yu, Luonan Chen, Kazuyuki Aihara

**Affiliations:** 1 Institute of Industrial Science, the University of Tokyo, Tokyo, Japan; 2 College of Statistics and Applied Mathematics, Anhui University of Finance and Economics, Bengbu, Anhui Province, China; 3 Key Laboratory of Systems Biology, CAS Center for Excellence in Molecular Cell Science, Innovation Center for Cell Signaling Network, Institute of Biochemistry and Cell Biology, Shanghai Institutes for Biological Sciences, Chinese Academy of Sciences, Shanghai, China; 4 School of Mathematics and Statistics, Shandong University at Weihai, Weihai, China; 5 School of Mathematics, South China University of Technology, Guangzhou, China; 6 School of Life Science and Technology, ShanghaiTech University, Shanghai, China; University of Virginia, UNITED STATES

## Abstract

Dynamic network biomarkers (DNB) can identify the critical state or tipping point of a disease, thereby predicting rather than diagnosing the disease. However, it is difficult to apply the DNB theory to clinical practice because evaluating DNB at the critical state required the data of multiple samples on each individual, which are generally not available, and thus limit the applicability of DNB. In this study, we developed a novel method, i.e., single-sample DNB (sDNB), to detect early-warning signals or critical states of diseases in individual patients with only a single sample for each patient, thus opening a new way to predict diseases in a personalized way. In contrast to the information of differential expressions used in traditional biomarkers to “diagnose disease”, sDNB is based on the information of differential associations, thereby having the ability to “predict disease” or “diagnose near-future disease”. Applying this method to datasets for influenza virus infection and cancer metastasis led to accurate identification of the critical states or correct prediction of the immediate diseases based on individual samples. We successfully identified the critical states or tipping points just before the appearance of disease symptoms for influenza virus infection and the onset of distant metastasis for individual patients with cancer, thereby demonstrating the effectiveness and efficiency of our method for quantifying critical states at the single-sample level.

## Author summary

The concept of dynamic network biomarkers (DNB) was proposed for detecting the critical state or tipping point of a complex disease (a pre-disease state immediately preceding the disease state), and has been applied to study the mechanism of cell fate decision and immune checkpoint blockade. But DNB cannot be used to identify the critical state or tipping point for a single patient because evaluating DNB for critical state required the data of multiple samples. The proposed method can identify the critical state of a complex disease for a single patient by implementing the concept of DNB. This method not only can be applied to detect the critical state or tipping point of a single sample, but also can be used to study the mechanism of complex disease at a single sample level. The ability of accurately and efficiently identifying the critical state for a single sample can benefit the development of personalized medicine.

This is a *PLOS Computational Biology* Methods paper

## Introduction

**Fig 1:**
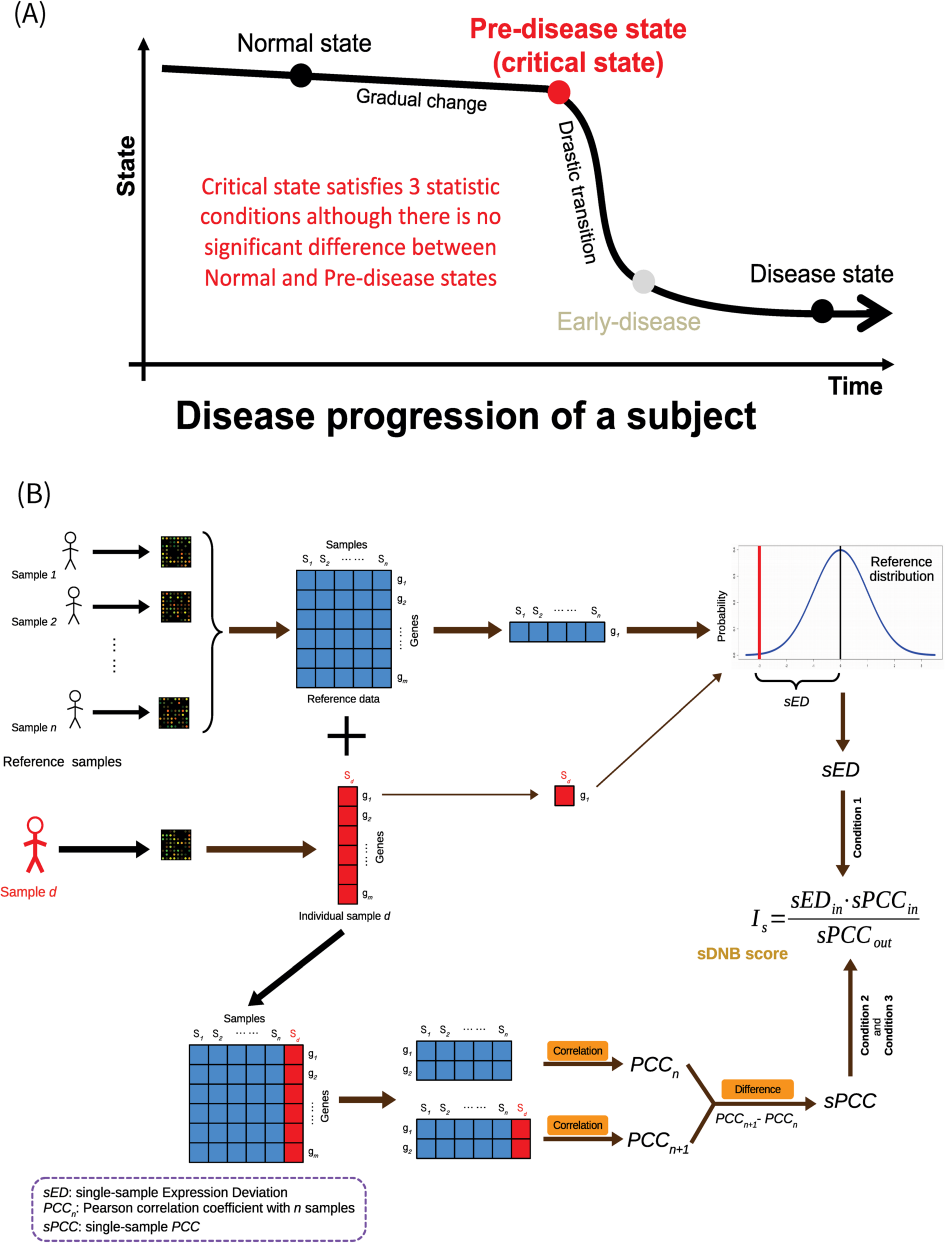
Disease progression and dynamic network biomarkers. (A) Three states during a disease progression. Clearly, there are significant differences between normal and disease states in terms of molecular expressions, and that is why traditional biomarkers can identify the disease state based on the differential information between them. But generally there is no significant difference between normal and pre-disease states, and thus traditional biomarkers may fail to detect the critical state for correctly predicting the disease. (B) Flowchart for calculating the composite index of single-sample dynamic network biomarkers (sDNB), which can detect the pre-disease state based on the three statistical conditions, rather than the differential expressions. Reference samples are required to produce the reference data. The distribution of every gene in terms of expression can be obtained from the reference samples, and the absolute value of the difference between a gene’s expression in an individual sample *d* and the average value of the gene’s expression in the reference samples is defined as the single-sample expression deviation (*sED*) of the gene for sample *d*. The Pearson correlation coefficient (*PCC*) between two genes in the reference samples is defined as *PCC*_*n*_. After the expression profile of sample *d* is added to the reference samples, the new correlation coefficient between the two genes can be obtained as *PCC*_*n+1*_. The difference between *PCC*_*n*_ and *PCC*_*n+1*_ can be regarded as the single-sample *PCC* (*sPCC*) between the two genes for sample *d*. The detail computation procedure of the sDNB score *I*_*s*_ is described in Fig 2.

Biomarkers, which are indicators of physiological states for living things, are commonly used to examine organ functions or disease states in biology or medicine. Generally, complex disease progression can be divided into three states, i.e., normal, pre-disease and disease (Fig 1A) [1, 2], where the pre-disease state is the critical state or tipping point from the normal to disease state and is also the limit of the normal state just before the critical transition to the disease state. The pre-disease state is usually considered to be reversible to a normal state if appropriately treated [1, 2], in contrast to disease states such as cancer and diabetes that are generally difficult to return to the normal state. Thus, the pre-disease state is a crucial state during the disease progression. However, it is hard to be identified by traditional biomarkers due to its similarities to the normal state in phenotypes and expressions, i.e., there are generally no significant differences between the normal and pre-disease states in terms of gene or protein expressions. Most traditional biomarkers are based on information about differential expressions, and thus mainly aim to distinguish the disease state from the normal state rather than diagnosing the pre-disease state before the onset of a disease. Therefore, identifying the pre-disease state, or the early-warning signals of the disease state, is an important challenge in medicine, and is not only beneficial for the early diagnosis and treatment of complex diseases but also provides dynamical insights into the molecular mechanism of complex diseases at a network level. To tackle this problem, the new concept of dynamic network biomarker (DNB) with its three statistical conditions was proposed to detect early-warning signals before disease onset at the molecular network level, and was applied to the analyses on various diseases [1–3]. Recently, our DNB model has also been adopted by many groups, successfully identifying the tipping points of cell fate decision [4, 5] and further studying immune checkpoint blockade [6]. DNB theory suggests that a molecular module or DNB will appear at the critical state (the pre-disease state or tipping point), and that this can be taken as an early-warning signal during the disease progression from normal to disease onset [1–3]. Specifically, we can theoretically prove that when a biological system from a normal state approaches the critical state, a DNB module or a group of molecules (or variables) appear and satisfy the following three statistic conditions [1–3]:

[Condition 1] deviation for molecules inside the module (*SD*_*in*_: standard deviation) drastically increases,
[Condition 2] correlation between molecules inside the module (*PCC*_*in*_: Pearson correlation coefficient in absolute value) rapidly increases, and
[Condition 3] correlation between molecules inside and outside the modules (*PCC*_*out*_: Pearson correlation coefficient in absolute value) rapidly decreases [1–3].

The above three statistical conditions are generic features of critical states, which hold for general biological systems regardless of their detail differences. Thus, we can simplify these three conditions as an index *I*_*m*_ of Eq (1) to evaluate the DNB module and detect the early-warning signals or the critical state in multiple samples as follows:

$$I_{m}=\frac{SD_{in}\cdot PCC_{in}}{PCC_{out}}$$ (1)

where *SD*_*in*_ is the average standard deviation (*SD*) for molecules inside the DNB module, *PCC*_*in*_ is the average Pearson correlation coefficient (*PCC*) in absolute value for molecules inside the module, and *PCC*_*out*_ is the average *PCC* in absolute value for molecules between the inner and outer molecules of the module. Clearly based on the three conditions, *I*_*m*_ will drastically increase when the biological system approaches the critical state, and thus it can signal the immediate disease state or predict disease state. In contrast to the information of differential expressions widely used in traditional biomarkers to “diagnose disease”, DNB is based on the information of differential associations, thereby having the ability to “predict disease” or “diagnose the un-occurred disease”.

DNB is a type of network biomarker, which can be used for the diagnosis of a pre-disease state rather than a disease state. In other words, DNB can be used for early diagnosis of a disease or to distinguish the pre-disease state from the normal state in complex diseases. According to the above three statistical conditions, DNB is clearly independent of differential expressions and is based on higher-order statistical information (i.e., the second-order moments) rather than the first-order statistics (i.e., the mean values or first-order moments) used for traditional molecular biomarkers. However, although the theory of DNB can ensure the recognition of early-warning signals of complex diseases, it requires multiple data samples to evaluate the three statistical conditions of DNB, which limits its application to clinical practice because multiple samples for each individual are generally not available. Here, by exploiting the high-dimensional information of the observed data (e.g., omics data) and its differential distribution (i.e., volcano distribution) [7], we propose a novel method to identify DNB modules and critical states on a single-sample basis. In other words, this single-sample DNB (sDNB) method based on the volcano distribution can detect the critical state using only a single sample, thus having a wide range of applications on biology and medicine. One influenza virus infection dataset and three cancer metastasis datasets were used to validate the effectiveness and efficiency of this method for quantifying critical states or tipping points on a single-sample basis. For the influenza virus infection dataset, we obtained individual sDNBs, which identified the critical states or early-warning signals just before the onset of disease symptoms (i.e., the clinical symptoms of influenza, such as fever, runny nose, and sore throat) [8]; no signal was detected in asymptomatic samples that showed few to no overt clinical symptoms of influenza [8], with the exception of one false-positive sample. For the cancer metastasis datasets, our method detected the critical states before the distant metastasis stage (stage IV) in stage IIIB and stage III cancer samples. Functional enrichment analysis showed that the functions of sDNB genes are consistent with the phenotype of viral infection for influenza virus infection and cancer processes for cancer metastasis. The analyses of real data also provided biological insights into the molecular mechanisms of the critical transitions from the perspectives of both molecules and networks for these complex diseases.

sDNB is the first such a method to predict the pre-disease state or quantify the tipping point based on only one sample. Note that, completely different from the traditional classification or machine learning methods which require a large number of case/control samples (for supervised or unsupervised learning) to obtain the predictor (overlearning problem, population-based predictor), sDNB is a model-free method and does not require any learning process on sample data. In other words, the predictor “sDNB” is constructed by the three statistical conditions for each specific sample that are actually based on the essential dynamical features of critical states for general biological systems, and thus inherently has no overlearning problem (even for a small sample size) and in particular is an individual-based predictor.

## Methods

To identify the critical state using DNB from a single sample, a control sample set is required as a reference. The information from the single sample can be extracted by comparing it with the reference samples [8]. In general, normal samples can be used as the reference samples and their expression profiles as the reference dataset.

### Dataset

Four datasets, including dataset GSE30550 [8] from the GEO database (http://www.ncbi.nlm.nih.gov/geo/) and datasets on lung adenocarcinoma (LUAD), stomach adenocarcinoma (STAD), and thyroid carcinoma (THCA) from the TCGA database (http://cancergenome.nih.gov), were used to validate the sDNB method. Dataset GSE30550 was normalized by the robust multi-array average (RMA) method, and the IDs of probe sets were mapped to the gene symbols. Probe sets without corresponding gene symbols were not considered in this study. The LUAD, STAD, and THCA datasets contained RNA-Seq data and included both tumor and tumor-adjacent samples. The tumor samples were divided into different stages based on clinical (stage) information from TCGA, while samples without stage information were ignored.

### Score of sDNB for quantifying critical states

We estimated sDNB score by using the three statistical conditions of DNB. Based on a number of reference samples (e.g., the dataset of normal or control samples), we can obtain expression distribution for each gene as its reference distribution. The expression of the gene in a new sample *d* (e.g., a case sample for statistical testing) can be compared with its reference distribution to estimate the deviation of its expression from the reference samples (*n* samples). The expression deviation of a gene in the new sample can be expressed as the distance from the expectation of its reference distribution (Fig 1B). Specifically, as Condition 1, the expression deviation of a gene in a single sample against *n* reference samples, i.e., the single-sample Expression Deviation (*sED)* for gene *x*, can be defined as

$$sED(x)=\left|x-\bar{x}\right|$$ (2)

where *x* is the expression of gene *x* in the new single sample, and $\bar{x}$ is the average expression value of gene *x* in the reference samples.

We assumed the number of samples in reference data to be *n*, and thus the Pearson correlation coefficient (*PCC*) between two genes (x, y) in the reference sample data can be calculated as

$$PCC_{n}(x,y)=\frac{\sum_{i=1}^{n}\left(x_{i}-\bar{x}\right)\left(y_{i}-\bar{y}\right)}{\sqrt{\sum_{i=1}^{n}{\left(x_{i}-\bar{x}\right)}^{2}\sum_{i=1}^{n}{\left(y_{i}-\bar{y}\right)}^{2}}}$$ (3)

where *x*_*i*_ and *y*_*i*_ are the expressions of gene *x* and gene *y* for the *i*th sample in the reference samples, respectively. $\bar{x}$ and $\bar{y}$ are the average gene expressions of gene *x* and gene *y* in the reference samples, respectively.

*PCC*_*n*_*(x*, *y)* is the correlation between two genes (x, y) in *n* reference samples. After a new single sample is added to the reference samples (Fig 1), the new *PCC* can be calculated for the two genes by Eq (3) based on total *n + 1* samples (i.e., *n* reference samples and one new sample *d*), i.e., *PCC*_*n+1*_*(x*,*y)*. The difference between *PCC*_*n+1*_ and *PCC*_*n*_ for the two genes is caused by the new single sample added to the reference data (Fig 1B), and hence it characterizes the specific correlation information of this single sample against the reference samples. Thus, as the conditions 2–3, we define the single-sample *PCC* (i.e., *sPCC*) of the two specific genes (x, y) against *n* reference samples as follows [7]:

$$sPCC(x,y)=PCC_{n+1}(x,y)-PCC_{n}(x,y)$$ (4)

which is clearly a differential PCC between *n+1* samples and *n* samples. Since *PCC* follows the normal distribution, *sPCC* in Eq (4) follows the differential normal distribution with *n* common samples. The significance of *sPCC* can be evaluable by a statistical method or the volcano distribution, i.e., the single-sample network theory [7]. Specifically, the “Z” score can be calculated for each *sPCC* by Eq (5), and the *p*-value of each *sPCC* can be approximately obtained from the standard normal cumulative distribution based on the “Z” score. Hence, the significance of *sPCC(x*, *y)* for any two genes (x, y) can be evaluated by the *p*-value of the “Z” score from Eq (5) as follows:

$$Z(x,y)=\frac{sPCC(x,y)}{\left(1-PCC_{n}^{2}(x,y)\right)/\left(n-1\right)}$$ (5)

where *PCC*_*n*_*(x*, *y)* is the Pearson correlation coefficient between two genes (x, y) in the reference samples, *n* is the sample size of the reference data, *sPCC(x*, *y)* is the differential *PCC* between *PCC*_*n+1*_ and *PCC*_*n*_ for the two genes (x, y) in Eq (4), Z(x, y) is the “Z” score of the Z-test for the two genes (x, y), and the *p-*value can be calculated as the standard normal cumulative distribution function [9]. Note that we can directly evaluate the significance of Z based on the volcano distribution without approximation [7]. Also we can directly use Z(x,y) as the normalized differential PCC for the single sample without the statistical test. Actually, such an implementation can be considered as a new transformation from gene expression data to the correlation-like data for each sample.

Therefore, based on the significant *sPCCs* of all pairs of genes or molecules, we can determine their corresponding network, which is perturbed by the single sample, and this network in turn also characterizes this single sample [7]. Based on the three statistical conditions of DNB shown in Eq (1), the composite index or score *I*_*s*_ of DNB (with *K* genes or molecules) to identify the pre-disease state for a single sample (single-sample DNB: sDNB) from all *sED* and *sPCC* in a module can be expressed as

$$I_{s}=\frac{sED_{in}\cdot sPCC_{in}}{sPCC_{out}}$$ (6)

where *I*_*s*_ is the score for sDNB based on the single sample. Here, *sED*_*in*_ indicates the average expression deviation of all *K* genes in the sDNB module relative to the reference samples, *sPCC*_*in*_ is the average correlation (for all *K*^*2*^ pairs) among whole genes in the sDNB module in absolute value, and *sPCC*_*out*_ is the average correlation (for all pairs) between the *K* inner and (*n-K*) outer genes of the sDNB module in absolute value. Next, we describe how to determine the *K* genes or molecules of DNB module, which has the highest score of *I*_*s*_. The detail formulation or derivation of Eq (6) is given in S1 Text, where *I*_*s*_ is shown to approximately represent the DNB score of the single sample.

### Algorithm to identify sDNB module for each sample

**Fig 2:**
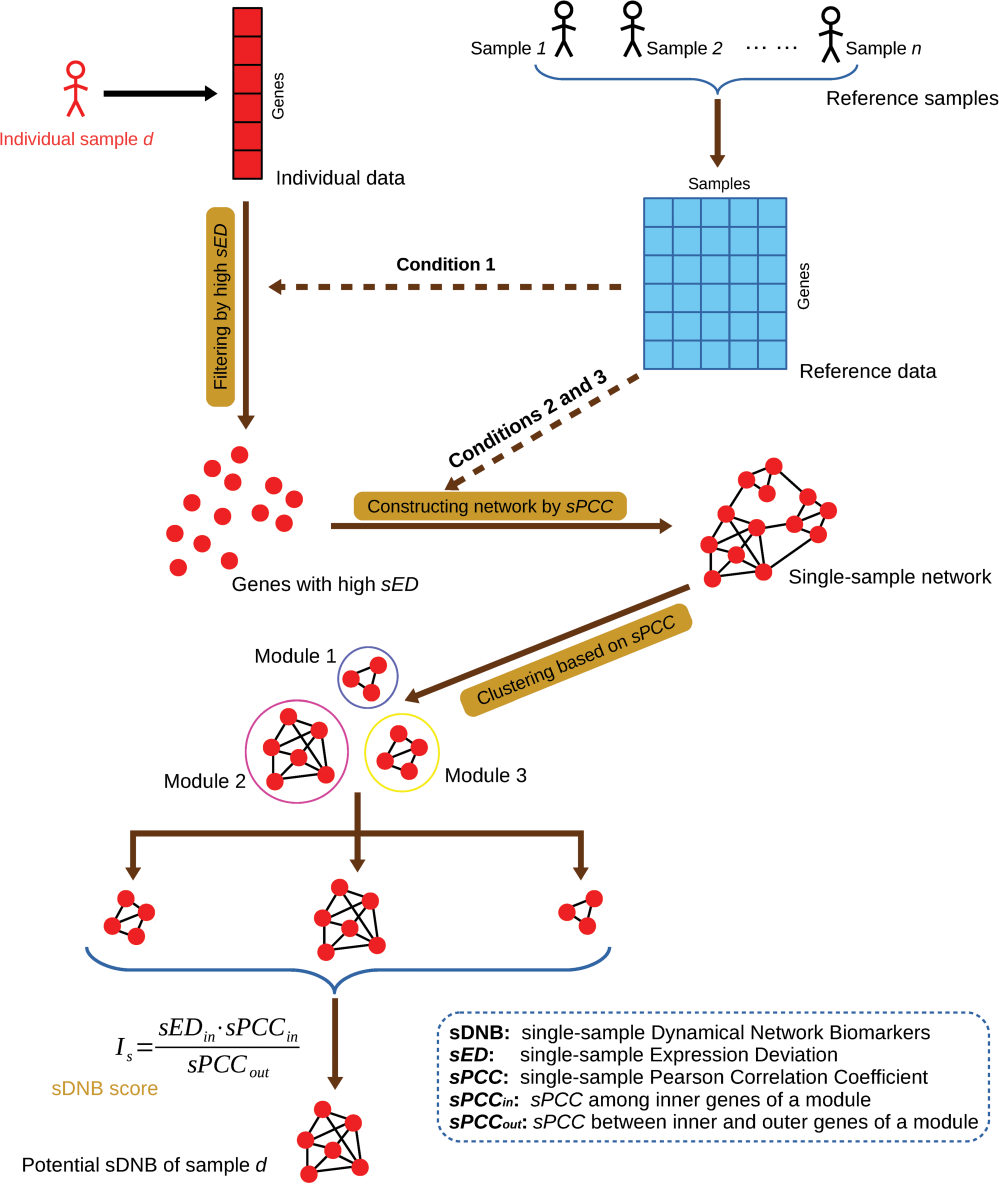
Flowchart of the algorithm for identifying potential sDNB in a single sample. *sED* and *sPCC* can be calculated by the method shown in Fig 1B. The hierarchical clustering algorithm was employed in the clustering process, and the value of 2 minus the absolute value of *sPCC* was used as the distance between genes for the hierarchical clustering algorithm.

A potential sDNB module can be detected for every single sample against the reference data (Fig 2). Generally, each individual or sample has a number of modules, and the module with the highest score *I*_*s*_ is the candidate DNB module for this specific sample. For a specific sample, we have the following algorithm to estimate the candidate DNB module.

1. Screening genes with high deviations (Condition 1): *sED* can be calculated for a specific sample (or a test sample) and every gene against the reference samples using Eq (2). The genes with high *sED* are chosen for usage in the following steps, and the genes with low *sED* are ignored. In such a way, we only keep a part of all *m* genes or molecules with high *sED* in this sample. The reference samples are required to be more than 8 samples, and can overlap with the test samples. But once they are chosen, the reference samples should not be changed for the computation of all test samples.
2. Screening genes with high differential *PCC* (Conditions 2–3): *sPCC* is calculated for the specific sample and any gene pair in high *sED* genes against the reference samples by Eq (4). The significance of every *sPCC* can then be evaluated by Eq (5). An edge is linked between two genes if their *sPCC* is significant. A network, which we term a single-sample network, is constructed by identifying all of the edges with significant *sPCC*s among the high *sED* genes for the single sample.
3. Decomposing a network into multiple modules: Hierarchical clustering is employed to decompose the single sample network into multiple modules based on the *sPCCs*, and 2 − |*sPCC*| is used as the distance for hierarchical clustering because the numerical value of *sPCC* is the range [–2, 2]. Note that the minimum or single-linkage clustering was used as the linkage criterion of the hierarchical clustering in this paper.
4. Choosing sDNB module with the highest score *I*_*s*_ (conditions 1–3): Eq (6) is used to estimate every module, and the module with the maximal score from Eq (6) is regarded as the potential sDNB module for this sample. If this score is significantly high, this sample is considered in the critical state based on DNB theory and this module is the DNB module.

With such an algorithm, we can get the candidate sDNB for each sample one by one. If the sDNB for a sample during a biological process or disease progression has the highest score among all samples, this sample is considered to be in the critical state, and the corresponding period is also the critical period.

### Functional analysis of sDNB

Functional annotations were performed by searching the NCBI gene database (http://www.ncbi.nlm.nih.gov/gene). The enrichment analyses were separately obtained using web service tools from the Gene Ontology Consortium (GOC, http://geneontology.org) and g:Profiler (http://biit.cs.ut.ee/gprofiler/) and client software from INGENUITY IPA (http://www.ingenuity.com/products/ipa).

## Results

### Validation data and reference data

In this study, four datasets, GSE30550 from the GEO database (http://www.ncbi.nlm.nih.gov/geo/) and LUAD, STAD, and THCA from the TCGA database (http://cancergenome.nih.gov), were chosen to validate the effectiveness of this method of quantifying the critical states of diseases.

Dataset GSE30550 comprises expression profiles of humans with influenza virus infection. It contains data from 17 healthy adults who were inoculated with live influenza virus H3N2 and gene expression profiles for the 17 adults at 16 time points (-24, 0, 5, 15, 21, 29, 36, 45, 53, 60, 69, 77, 84, 93, 101, and 108 hours) by microarray (S1 Fig), i.e., there are 17 samples at each time point, corresponding to the 17 adults, respectively. Nine of the 17 adults developed disease symptoms of influenza, and the other eight were asymptomatic (S1 Fig). There are 11,961 probe sets and 17 samples in the original GSE30550 dataset, and 11,619 gene symbols were mapped from the ID of the probe sets. The gene expression values of the probe sets mapped to the same gene were calculated by an averaging operation. The gene expression profiles at -24 h (24 h before inoculating) were deemed as the normal states of the samples without virus inoculation, and the profiles of 16 samples (the data on sample 13 was lost at -24 h) at -24 h were chosen as the reference dataset or reference samples (S1 Fig).

From the LUAD (lung cancer), STAD (stomach cancer), and THCA (thyroid cancer) datasets, 459 tumor samples and 58 tumor-adjacent samples were obtained for LUAD. The tumor samples were grouped into seven stages (stage IA, IB, IIA, IIB, IIIA, IIIB, and IV) of lung cancer (Table 1 and S1 Table). One hundred fifty-six tumor samples and 33 tumor-adjacent samples were obtained for STAD. The tumor samples were grouped into seven stages (stage IA, IB, IIA, IIB, IIIA, IIIB, and IV) of stomach cancer (Table 1 and S1 Table). Three hundred fifty-seven tumor samples and 58 tumor-adjacent samples were obtained for THCA. The tumor samples were grouped into four stages (stage I, II, III, and IV) of thyroid cancer (Table 1 and S1 Table). The tumor-adjacent samples were considered as normal controls and were used as reference samples in this study.

**Table 1:** The number of tumor samples within each stage in the cancer dataset from TCGA.

		LUAD	STAD	THCA
**Stage I**	**Stage IA**	106	9	218
	**Stage IB**	124	18	
**Stage II**	**Stage IIA**	39	23	44
	**Stage IIB**	59	29	
**Stage III**	**Stage IIIA**	62	27	82
	**Stage IIIB**	10	20	
**Stage IV**	**Stage IV**	21	15	13
**TA samples**	**TA samples**	58	33	58

TA samples are tumor-adjacent samples, and were used as the reference dataset in this study.

### sDNB and disease prediction in influenza virus infection

**Fig 3:**
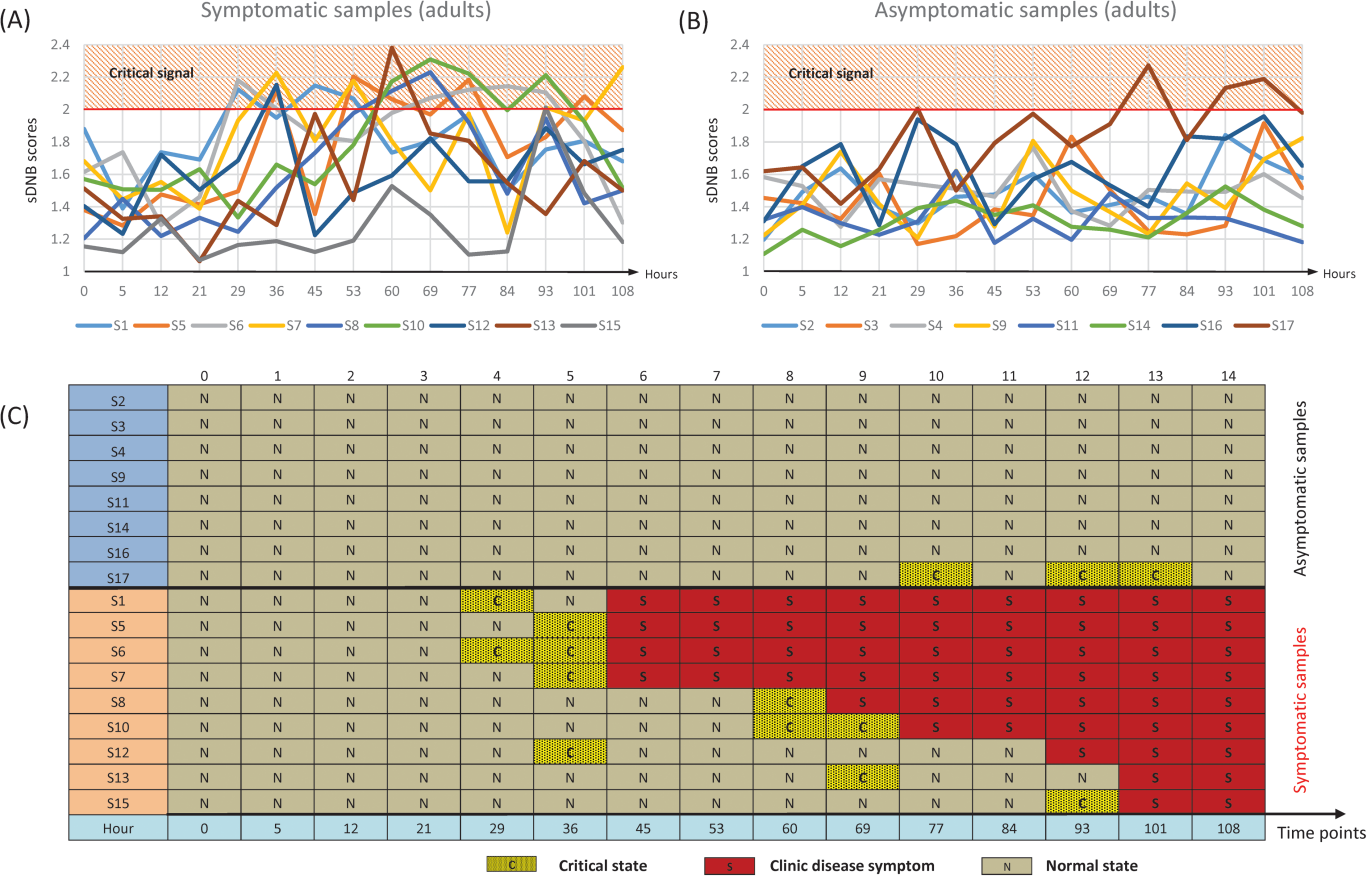
Quantifying the critical states for the influenza virus infection data [8]. (A) Line chart for early-warning signals in all symptomatic adults. (B) Line chart for early-warning signals in all asymptomatic adults. (C) Table of sDNB diagnoses and clinical diagnoses for all adults and samples.

The gene expression profiles of samples at time point −24 h (24 hours before inoculation) were chosen as reference samples, and totally 16 normal samples were included in the reference data. The candidate sDNB for each adult was identified by comparing the case sample with the reference samples. The threshold of s*PCC* was set to the *p-*value of 0.01 in the process of constructing the single-sample network (Fig 2), and the sDNB score was set to 2.0 for detecting the critical state, or early-warning signals, of the disease, or symptomatic, state for every sample. For all nine symptomatic samples, the scores *I*_*s*_ of their sDNB modules were significantly high before the disease state, and thus correctly signaled the imminent emergence of the disease state (before disease symptom appearance) (Fig 3A and 3C). In contrast, for the seven asymptomatic samples, no early-warning signal was detected based on the sDNB scores, but one asymptomatic sample (s17) exhibited false-positive early-warning signals at later time points (Fig 3B and 3C). Notably, all eight asymptomatic samples were Caucasian/White except sample s17 (Indian) who may have had a different threshold. Note that most of the subjects (14 of 17 samples) in this dataset were Caucasian/White (S1 Fig). Another possibility is that sample s17 did reach the critical state but returned to the normal state without further disease progression.

For most adults (16 of 17 symptomatic and asymptomatic adults) in dataset GEO30550, our method could correctly detect critical states or early-warning signals before disease symptom appearance based on the sDNB scores (single samples). False-positive warning signals for only one adult appeared in the asymptomatic samples, possibly due to the causes described above.

The module size of sDNB in every symptomatic adult was different, e.g., there were 1553 sDNB genes for adult s7, 696 for adult s5 (S2 Table), and only 350 for adult s10, based on the same conditions (S2 Table). The average number of overlapping genes between any two sDNBs is approximately 37.8% (S4 Table). There were 25 overlapped genes among all nine sDNBs in the symptomatic samples (S3 Table). Functional annotations were done for these overlapped genes by searching NCBI for *Homo sapiens*, and results are shown in S3 Table.

### Functional analysis of sDNB in influenza virus infection

Enrichment analysis of the 25 overlapped genes among all 9 sDNBs of symptomatic samples (S3 Table) was performed using web services in Gene Ontology Consortium and g:Profiler and the client software of IPA.

The sDNB modules identified in the symptomatic samples are shown in S2 Table, and the overlapped genes among all sDNBs in symptomatic samples are shown in S3 Table. There were 25 genes in the overlapping of 9 sDNBs among all symptomatic samples, and the results of enrichment analysis for the 25 overlapped genes are shown in Table 2. The overlapped gene functions included some processes of response to virus, consistent with phenotypes of the nine samples infected by the influenza virus. Because the functions of the 25 overlapped genes were enriched to the processes of defense response, negative regulation to virus, or antivirus response (Table 2), it appears that the process of immunity or defense against the influenza virus may start in the immune system, and the immune systems of the nine symptomatic samples could not stop the further “invasion” of the influenza virus, resulting in the influenza phenotype. The time points identified by sDNBs may be the critical points of influenza virus “invasion” (defeating the immune system). The functional enrichment of the overlapped genes of all sDNBs is consistent with the phenotype of invasion of the influenza virus and the response of the immune system to defend the virus.

**Table 2:** The functional enrichment of the overlapped genes among sDNB for influenza virus infection.

Gene Ontology Consortium		g:Profiler		IPA	
enriched items	enriched *p* value	enriched items	enriched *p* value	enriched items	enriched *p* value
defense response to virus (GO:0051607)	1.12×10^−17^	defense response to virus (GO:0051607)	1.31×10^−15^	antiviral response	1.63×10^−11^
response to virus (GO:0009615)	2.26×10^−15^	response to virus (GO:009615)	3.1×10^−14^	Viral Infection	2.66×10^−10^
defense response to other organism (GO:0098542)	6.07×10^−13^	defense response to other organism (GO:0098542)	3.28×10^−12^	replication of virus	5.41×10^−09^
response to external biotic stimulus (GO:0043207)	5.67×10^−11^	cellular response to type I interferon (GO:0071357)	1.62×10^−10^	replication of RNA virus	5.98×10^−07^
immune response (GO:0006955)	8.33×10^−11^	immune response (GO:0006955)	1.72×10^−09^	replication of viral replicon	6.35×10^−07^

Nineteen of the 25 overlapped genes are reported to be related to virus response, and 6 of the 19 are associated with the influenza virus (S3 Table). Hence, most of the genes identified by sDNB may be potential target genes for further study of the mechanism of the interaction between the influenza virus and human beings in the future. Note that although the data of the 6 genes are common in all nine symptomatic subjects, they do not have sufficient information to detect the early-warning signal for each subject. Actually, for the diagnostic purpose, it is preferred to use all measured genes (e.g., 20000 genes), which include available information to identify sDNB for signaling the critical state of the disease progression of each subject.

### sDNB and critical states for tumor disease

Fifty-eight tumor-adjacent samples were taken as reference samples for LUAD (Table 1), 33 as reference samples for STAD (Table 1), and 58 as reference samples for THCA (Table 1). The potential sDNB for each sample was detected by the following method (Fig 2). The threshold of s*PCC* was set as the *p*-value of 0.01 to construct the single-sample network (Fig 2), and the module with the maximal score in each sample was regarded as the potential sDNB for this sample.

The progression and development of cancer can be divided into stages, such as stage I, stage II, stage III, and stage IV. Metastasis, the major cause of recurrence and death in cancer patients, is a complex interplay between malignant cancer cells and surrounding tumor microenvironments [10]. Stage IV is usually an advanced or metastatic cancer in which the tumor has spread or metastasized to other organs or parts of the human body [11, 12]. All of the samples were grouped into different cancer stages based on clinical information from the TCGA database. The index score *I*_*s*_ of each potential sDNB module was calculated for every single sample, and the average index score of every stage for sDNB was used to identify the critical state or quantify the early-warning signals for cancer metastasis.

**Fig 4:**
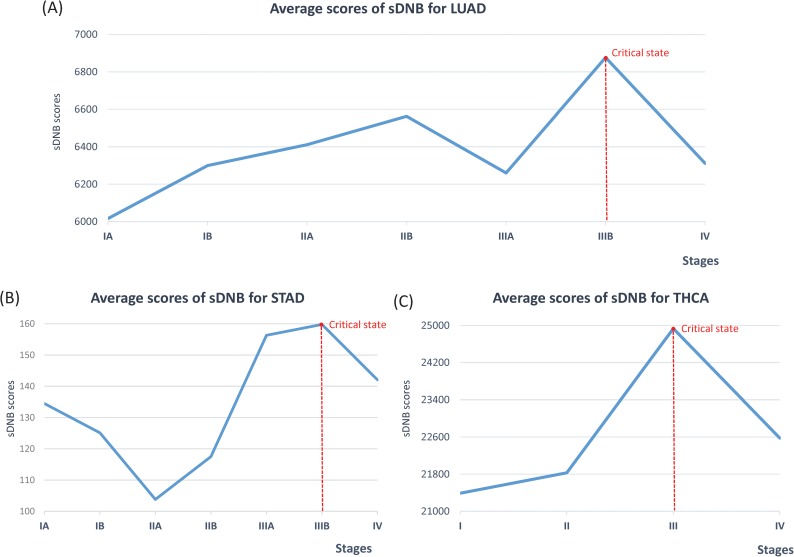
Quantifying the critical states for metastasis in three cancers: (A) LUAD, (B) STAD, and (C) THCA.

For LUAD, STAD, and THCA, all the peaks for the average sDNB score appeared before stage IV, which is the cancer metastasis stage (Fig 4), and these peaks were considered the early-warning signals for cancer metastasis.

There are seven stages (IA, IB, IIA, IIB, IIIA, IIIB, and IV) in the cancer progression of LUAD (S1 Table), and the maximal score of the average sDNB index was detected in stage IIIB (Fig 4A), which is the last stage before cancer metastasis. There were 10 samples of stage IIIB LUAD in TCGA (Table 1), with 10 sDNBs identified by our method (S8 Table). Thirty-three genes appearing in at least eight (80%) sDNBs were regarded to be related to the cancer metastasis of LUAD (S5 Table). Some genes in this list have been shown to be associated with the process of cancer metastasis. For instance, SRPK1 is regarded as the molecular determinant of tumor cell migration and cancer metastasis [13]. TOP2A is related to brain metastasis for non-small-cell lung cancer [14]. CDC25C is related to the metastasis of cancer [15, 16]. IQGAP3 is also related to cancer metastasis [17, 18]. PRAME is a cancer metastasis gene in uveal melanoma [19] and in lung cancer [20]. XRCC2 is related to the metastasis of colorectal cancer [21]. TUBB3 is related to breast cancer metastasis to the brain [22] and metastasis in pancreatic cancer [23]. HDGF is related to the regulation of cancer metastasis [24, 25], and especially to the metastasis of lung cancer [26, 27]. SPAG5 is related to the metastasis of prostate cancer [28]. The above genes have all been reported to be associated with cancer metastasis, and they might also regulate and/or provide early-warning signals for the cancer metastasis process in LUAD.

Functional enrichment showed that the common genes in at least 80% of sDNBs are identified as genes involved in the biological processes of the nuclear division, the mitotic cell cycle, the organelle fission, and so on (Table 3) by GOC (the gene ontology consortium) and g:Profiler, and these biological processes are associated with the progression of cancer. These common genes were also related to stage 4 non-small-cell lung carcinoma and metastatic non-small-cell lung cancer by functional enrichment in IPA (Table 3); this is consistent with our assumption, based on the DNB theory, about the critical state of tumor metastasis for non-small-cell lung cancer prior to stage IV.

**Table 3:** The functional enrichment of sDNB genes in at least 80% of samples for LUAD.

Gene Ontology Consortium		g:Profiler		IPA	
enriched items	enriched *p* value	enriched items	enriched *p* value	enriched items	enriched *p* value
nuclear division (GO:0000280)	3.15×10^−06^	nuclear division (GO:0000280)	1.31×10^−05^	mitosis of tumor cell lines	6.02×10^−05^
mitotic cell cycle (GO:0000278)	3.21×10^−06^	organelle fission (GO:0048285)	2.51×10^−05^	stage 4 non-small-cell lung carcinoma	2.98×10^−03^
organelle fission (GO:0048285)	5.54×10^−06^	mitotic cell cycle (GO:0000278)	4.37×10^−05^	growth of tumor	4.63×10^−03^
mitotic cell cycle process (GO:1903047)	1.96×10^−05^	mitotic cell cycle process (GO:1903047)	7.72×10^−05^	metastatic non-small-cell lung cancer	6.74×10^−03^
cell cycle (GO:0007049)	9.10×10^−05^	mitotic nuclear division (GO:0007067)	4.53×10^−04^	lung cancer	1.95×10^−02^

There are also seven stages (IA, IB, IIA, IIB, IIIA, IIIB, and IV) in the cancer progression of STAD (S1 Table), and the maximal score of the average sDNB index was detected in stage IIIB (Fig 4B), which is the last stage before cancer metastasis. There were 20 samples recorded as stage IIIB STAD in TCGA (Table 1), and 20 sDNBs were identified from these 20 samples (S9 Table). Eighteen genes appeared in at least 10 (50%) of the sDNBs and were considered to be related to the cancer metastasis of STAD (S6 Table). Some genes in this list have been reported to be associated with the process of cancer metastasis, e.g., COL11A1 has been identified as a remarkable biomarker for carcinoma progression and metastasis [29] in breast cancer [30] and serous ovarian cancer [31]. CST1-overexpressing cell lines exhibit increased metastasis in a mouse model [32, 33]. High expression of CST4 can promote bone metastasis *in vivo* [33]. CTHRC1 is upregulated and enhances the epithelial-mesenchymal transition of tumor cells to promote cancer invasion and metastasis in colorectal cancer [34–36] and melanoma [37]. ESM1 regulates cell growth and the metastatic process by activation of NF-κB in colorectal cancer [38]. The overexpression of FGF19 is significantly associated with tumor-distant metastasis in thyroid cancer [39]. High expression of IBSP is associated with bone metastasis in breast and prostate cancers [40, 41]. PRAME is also one of the 33 genes in the overlapped sDNB of LUAD [19, 20]. PRAME is a cancer metastasis gene involved in uveal melanoma [19] and lung cancer [20]. Wnt2 plays an important role in the metastasis of pancreatic cancer [42, 43]. The above genes are associated with cancer metastasis, and they may also regulate and/or provide early-warning signals for the cancer metastasis process in STAD.

Functional enrichment analysis showed that the common genes in at least 50% of sDNBs were involved in the biological processes of collagen catabolic process, multicellular organismal catabolic process, etc. (Table 4) according to GOC and g:Profiler, and these biological processes may characterize the alteration of tumor metabolism [44, 45]. These common genes were also related to the proliferation of cells, upper gastrointestinal tract cancer, and digestive organ tumor by functional enrichment from IPA (Table 4); this is consistent with our test, based on the DNB theory, for quantifying the critical states of tumor metastasis in gastric cancer.

**Table 4:** The functional enrichment of sDNB genes in at least 50% of samples for STAD.

Gene Ontology Consortium		g:Profiler		IPA	
enriched items	enriched *p* value	enriched items	enriched *p* value	enriched items	enriched *p* value
collagen catabolic process (GO:0030574)	2.19×10^−03^	collagen catabolic process (GO:0030574)	9.03×10^−04^	proliferation of cells	9.31×10^−04^
multicellular organismal catabolic process (GO:0044243)	3.13×10^−03^	multicellular organismal catabolic process (GO:0044243)	1.27×10^−03^	upper gastrointestinal tract cancer	1.47×10^−03^
collagen metabolic process (GO:0032963)	3.61×10^−03^	collagen metabolic process (GO:0032963)	5.51×10^−03^	digestive organ tumor	2.72×10^−03^
multicellular organismal metabolic process (GO:0044236)	4.75×10^−03^	multicellular organismal macromolecule metabolic process (GO:0044259)	6.65×10^−03^	digestive system caner	2.05×10^−02^
extracellular matrix disassembly (GO:0022617)	1.38×10^−02^	extracellular matrix disassembly	7.50×10^−03^	abdominal cancer	4.86×10^−02^

There are four stages (I, II, III, and IV) in the cancer progression of THCA (S1 Table). The peak score for the average sDNB index appeared in stage III (Fig 4C), which is also the last stage before cancer metastasis. There are 82 stage III samples (Table 1), from which 82 sDNBs were identified (S10 Table). Fifty-one genes appeared in at least 41 (50%) sDNBs and were considered to be related to cancer metastasis in THCA (S7 Table). Some genes in this list have been reported to be associated with the process of cancer metastasis. In particular, the expression of CITED1 is correlated with lymph node metastasis in patients with colorectal cancer [46]. CSF2 is one of the pivotal orchestrators of basal breast cancer growth and metastasis [47]. DPP4 shows positive metastatic activity in cancer cells [48]. FN1 plays a critical role in metastasis and is associated with advanced stages and higher metastatic potential in patients with renal cancer [49–51]. GRM4 is involved in the metastasis of osteosarcoma and affects the survival of osteosarcoma patients [52]. The expression of IGSF1 is associated with the invasion and metastasis of neoplasms by mediating homotypic and heterotypic intercellular adhesion and binding [53]. KLK10 plays essential roles in tumor invasion and metastasis in gastric cancer [54] and epithelial ovarian carcinomas [55]. The expression level of KLK7 is correlated with prognosis of liver metastasis in patients with colorectal cancer [56]. LAD1 is identified as a potential marker in renal cell cancer, showing univariate association with distinct metastasis [57]. Knockdown of LAM3 suppresses human lung cancer cell invasion and metastasis *in vitro* and *in vivo* [58]. LIPH is related to distant metastasis in breast cancer [59]. PROS1 can lead to regulation of local invasion and metastasis [60]. Enhanced SERPINA1 expression is significantly associated with invasion and metastasis in gastric cancer [61]. SLC34A2 strongly inhibits tumor growth and metastasis ability in non-small-cell lung cancer [62]. TENM1 is related to tumor metastasis in prolactin pituitary tumors [63]. TMPRSS4 mediates tumor cell invasion, migration, and metastasis [64]. The above genes are associated with cancer metastasis and might also regulate the critical state in the cancer metastasis process in THCA.

Functional enrichment showed that the common genes in at least 50% of the sDNBs are associated with thyroid cancer, papillary thyroid cancer, thyroid gland tumor, etc. (Table 5) according to IPA, which is consistent with the test for thyroid cancer. We also estimated the significance of sDNB to correctly signal the critical state (Stage III) for thyroid cancer. We first randomly picked up 82 samples from all THCA samples (see Table 1), and calculated their average score of sDNB. Then, the average score of sDNB for the random samples was compared with that of the 82 samples in Stage III. Such a random sampling was repeated 10000 times. The probability that the average sDNB score of the random samples is greater than that of all the samples in Stage III is regarded as the statistical significance for the identification of disease deterioration, and actually the p-value of the statistical significance is 0.0318 in THCA.

**Table 5:** The functional enrichment of sDNB genes in at least 50% of samples for THCA.

IPA Analysis	
enriched items	enriched *p* value
thyroid cancer	1.53×10^−05^
papillary thyroid cancer	3.19×10^−03^
thyroid gland tumor	4.17×10^−03^
invasive papillary thyroid carcinoma	9.47×10^−03^
quantity of thyroid hormone	1.14×10^−02^

## Discussion

In this study, by exploiting the high-dimensional information of the observed data and the volcano distribution of differential networks, a new method was proposed to identify tipping points or critical states (which appear just before the disease state) based on single-sample DNB (sDNB). In contrast to the information of differential expressions used in traditional biomarkers to diagnose disease, sDNB is based on the information of differential associations, thereby having the ability to predict disease or “diagnose the un-occurred disease”. This method was applied to quantify the early-warning signals for the process of influenza virus infection and cancer metastasis on a single-sample basis. The results for the influenza virus infection show that high sDNB scores indeed signaled the imminent emergence of disease symptoms (at least 8 hours before their appearance) for every symptomatic sample, and there were no significant high scores for asymptomatic samples with the exception of adult s17. A potential explanation for this false-positive result on adult s17 is that this asymptomatic adult was the only non-Caucasian/White subject among the asymptomatic adults, and may thus have had a different threshold. Another possibility is that adult s17 did reach the critical state but recovered to the normal state before further deterioration into the disease state, thereby causing a significant signal.

This method is also robust for quantifying early-warning signals by identifying the sDNB. When the threshold of *sPCC* was set at the *p-*value of 0.05 to construct the single-sample network (Fig 2), there were large fluctuations in the samples of symptomatic adults approaching disease symptoms and small fluctuations in the samples of asymptomatic adults, with the exception of adult s17 (S2 Fig). When the threshold of the sDNB score was set to 1.6, we obtained similar early-warning signals for predicting influenza symptoms, as shown in Fig 3 and S2 Fig. There were 10 sDNBs (S11 Table) and 54 overlapped genes (S12 Table) among the sDNBs based on this threshold, and functional enrichment showed that these 54 genes can also characterize the virus infection response (S13 Table), similar to the results shown in Table 2. Hence, the threshold of *sPCC* is robust, i.e., it does not significantly affect the results, although the threshold of the sDNB score for detecting the critical states is an empirical value in this study. It is our important future work to identify the sDNB threshold in a systematic and efficient way.

The results for cancer metastasis showed that sDNBs could detect the critical state of cancer metastasis before stage IV that is the stage when cancer-distant metastasis occurs. In particular, for LUAD, the overlapped genes of sDNBs in stage IIIB could be enriched to the processes of stage 4 non-small-cell lung carcinoma and metastatic non-small-cell lung cancer by IPA (Table 3), indicating that the function of the sDNBs identified in stage IIIB is related to the metastasis of LUAD in stage IV and that sDNBs provide the early-warning signals that can be used to predict the onset of metastasis for LUAD before it occurs.

Note that sDNB is a model-free method, and does not requires the learning on sample data; it is completely different from the traditional classification or machine learning methods which are population-based predictors requiring a large number of case/control samples to train the model and eliminate the overlearning problem. In other words, sDNB is an individual-based predictor based on the three statistical conditions for each specific sample, and thus inherently has neither overlearning problem nor assumption on the model. Hence, even for the same disease, the composition of sDNB as well as the size of sDNB for each sample or individual may be different, but its *I*_*s*_ drastically increases whenever approaching the critical state. However, we use a unified threshold in this paper, on the composite index of sDNB or Eq (6), for determining the critical state, which is based on the whole disease samples.

The critical state is considered as a stage early reversible to the normal state. Thus, appropriate treatment for subjects in the critical state is considered much effective in contrast to the subjects in the disease state. However, how to make such a treatment is beyond the scope of this work, and will be a future topic. In addition, theoretically, any omics data (e.g., transcriptomic data, proteomics data, or metabolomics data) which can dynamically reflect the change of the disease progression, can be used to detect the critical state or tipping point. Thus, depending on the disease type, we may choose an appropriate type of the omics data. With current high-throughput technologies, generally RNAs can be quantified in a relatively stable way in contrast to proteins and metabolites. Therefore, the transcriptomic data (e.g. RNA-Seq or microarray) are effective for sDNB identification from the computational viewpoint, although metabolomics and proteomics data can also be used to identify the critical state.

In summary, the method described in this paper developed a novel method, sDNB, which is the first such a method to predict disease state based only on a single sample, opening a new way to quantify the critical state of diseases in individual patients. Thus, the method can be directly applied not only to personalized pre-disease diagnosis but also to the molecular mechanism analysis of disease progression at the network level. In a similar way, sDNB could also be used to detect the tipping points or critical states of many nonlinear biological processes, such as cellular differentiation and cellular proliferation [4–6].

## Supporting information

S1 Fig Clinical information for all samples in the influenza virus infection data. (PDF)

S2 Fig Quantifying the critical states for the influenza virus infection data with a different threshold. (A) Line chart for early-warning signals in all symptomatic adults. (B) Line chart for early-warning signals in all asymptomatic adults. (C) Table of sDNB diagnoses and clinical diagnoses for all adults and samples.
(PDF)

S1 Table The stage distribution for the tumor samples of lung adenocarcinoma (LUAD), stomach adenocarcinoma (STAD) and thyroid carcinoma (THCA) from TCGA. (XLSX)

S2 Table sDNB and early-warning signals based on single sample. (XLSX)

S3 Table The overlapped genes among sDNB. (XLSX)

S4 Table The ratio of overlapped genes any between two sDNB. (XLSX)

S5 Table The genes of sDNB repeated emergence in at least 80% samples for LUAD. (XLSX)

S6 Table The genes of sDNB repeated emergence in at least 50% samples for STAD. (XLSX)

S7 Table The genes of sDNB repeated emergence in at least 50% samples for THCA. (XLSX)

S8 Table The genes of sDNB of every sample in stage IIIB for LUAD. (XLSX)

S9 Table The genes of sDNB of every sample in stage IIIB for STAD. (XLSX)

S10 Table The genes of sDNB of every sample in stage III for THCA. (XLSX)

S11 Table sDNB and early-warning signals based on the other threshold. (XLSX)

S12 Table The overlapped genes among the sDNB with p value of sPCC 0.05 and score of sDNB 1.6. (XLSX)

S13 Table The functional enrichment of the 54 overlapped genes among sDNB with p value of sPCC 0.05 and score of sDNB 1.6. (XLSX)

S1 Text Deriving a criterion of single-sample dynamic network biomarkers. (DOC)
