# Supplementary material for: Quantifying critical states of complex diseases using single-sample dynamic network biomarkers
Source: PLoS Comput Biol. 2017 Jul 5;13(7):e1005633. doi: 10.1371/journal.pcbi.1005633 (PMC5517040; doi:10.1371/journal.pcbi.1005633)
Supplement: S1 Fig — (PDF) [file pcbi.1005633.s001.pdf]

Reference data

Clinic symptom during the progression of influenza virus infection for 17 samples

17 samples in influenza virus infection experiment

S2	0	0	0	0	0	0	0	0	0	0	0	0	0	0	0	0	Caucasian/White
S3	0	0	0	0	0	0	0	0	0	0	0	0	0	0	0	0	Caucasian/White
S4	0	0	0	0	0	0	0	0	0	0	0	0	0	0	0	0	Caucasian/White
S9	0	0	0	0	0	0	0	0	0	0	0	0	0	0	0	0	Caucasian/White
S11	0	0	0	0	0	0	0	0	0	0	0	0	0	0	0	0	Caucasian/White
S14	0	0	0	0	0	0	0	0	0	0	0	0	0	0	0	0	Caucasian/White
S16	0	0	0	0	0	0	0	0	0	0	0	0	0	0	0	0	Caucasian/White
S17	0	0	0	0	0	0	0	0	0	0	0	0	0	0	0	0	Indian Subcontinent
S1	0	0	0	0	0	0	0	1	1	1	1	1	1	1	1	1	Black/Africa
S5	0	0	0	0	0	0	0	1	1	1	1	1	1	1	1	1	Caucasian/White
S6	0	0	0	0	0	0	0	1	1	1	1	1	1	1	1	1	Mixed Ethnicity
S7	0	0	0	0	0	0	0	1	1	1	1	1	1	1	1	1	Caucasian/White
S8	0	0	0	0	0	0	0	0	0	0	1	1	1	1	1	1	Caucasian/White
S10	0	0	0	0	0	0	0	0	0	0	0	1	1	1	1	1	Caucasian/White
S12	0	0	0	0	0	0	0	0	0	0	0	0	0	1	1	1	Caucasian/White
S13	0	0	0	0	0	0	0	0	0	0	0	0	0	0	1	1	Caucasian/White
S15	0	0	0	0	0	0	0	0	0	0	0	0	0	0	1	1	Caucasian/White
Time point	-24h	0h	5h	12h	21h	29h	36h	45h	53h	60h	69h	77h	84h	93h	101h	108h	Race

8 asymptomatic samples

9 symptomatic samples

Virus infection point

0 Non-symptom

1 Symptom
