# Supplementary figures and images for: Quantifying critical states of complex diseases using single-sample dynamic network biomarkers
Source: PLoS Comput Biol. 2017 Jul 5;13(7):e1005633. doi: 10.1371/journal.pcbi.1005633 (PMC5517040; doi:10.1371/journal.pcbi.1005633)

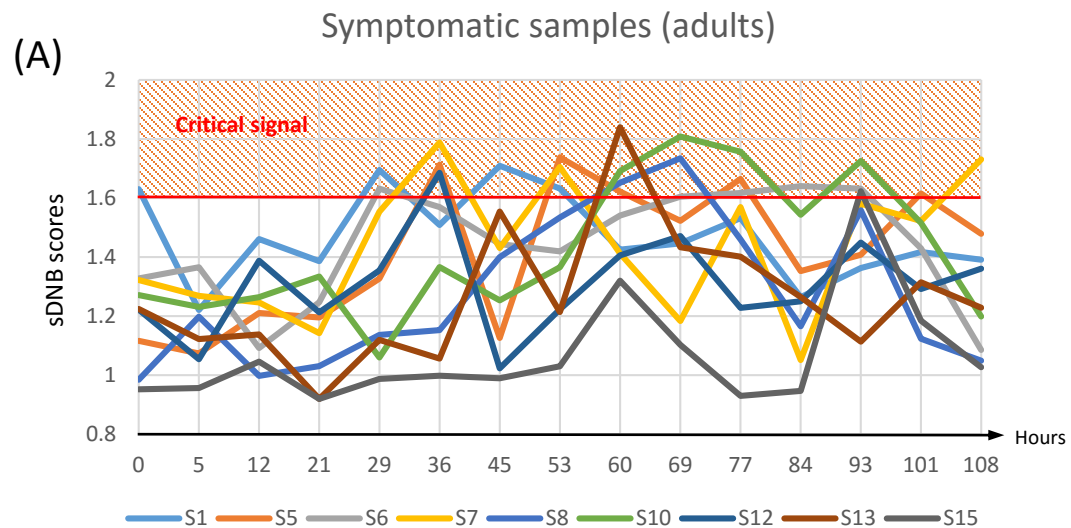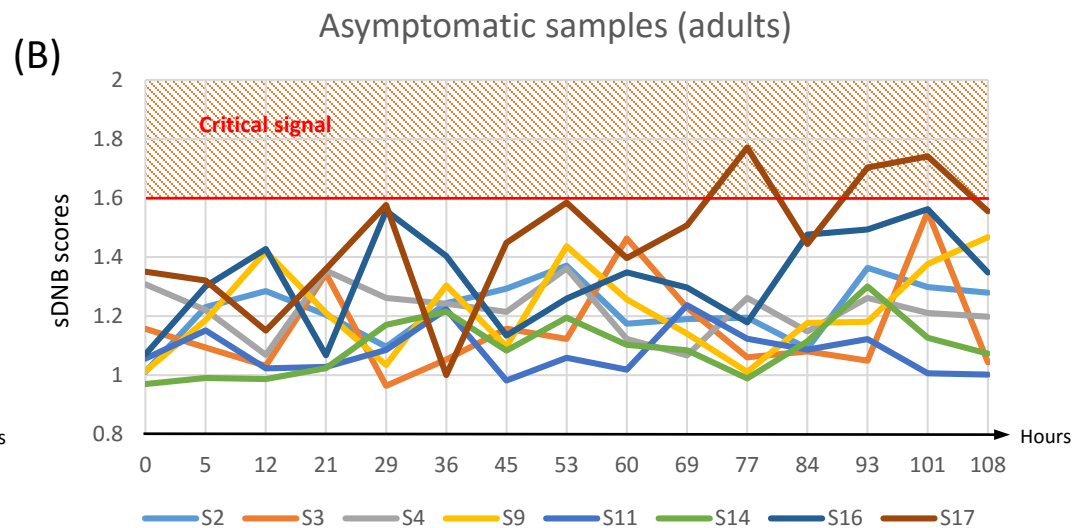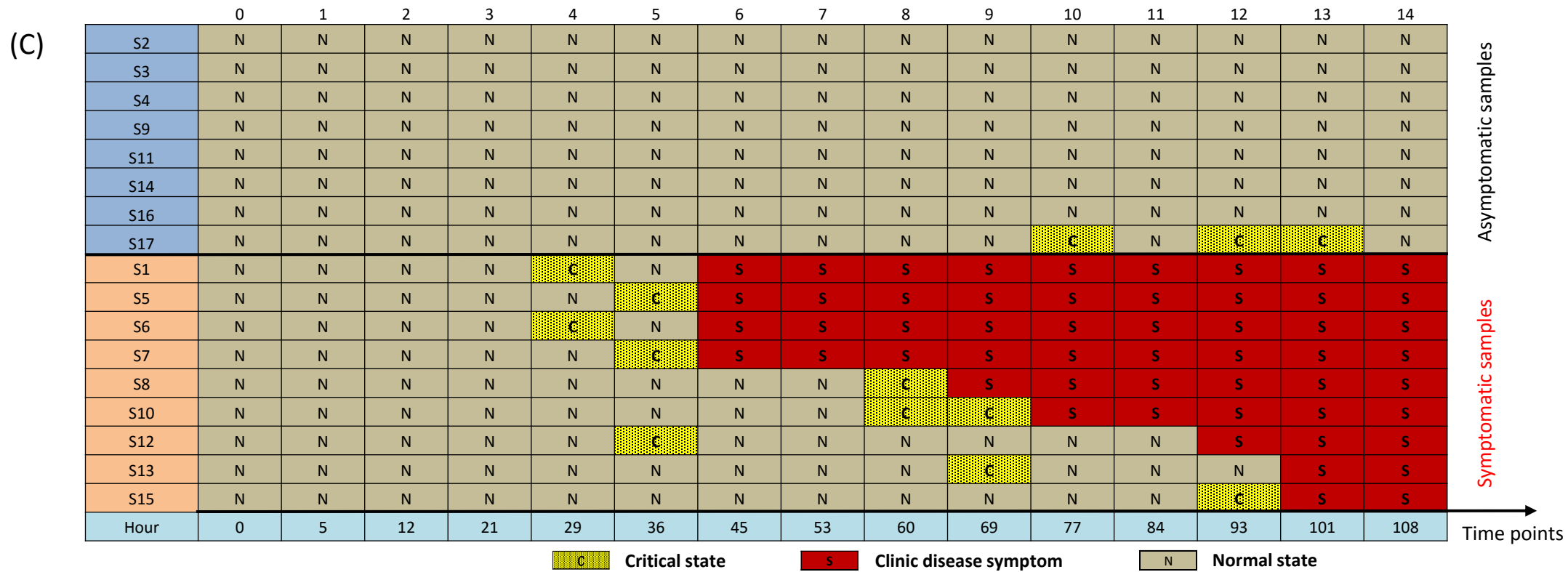

Supplement: S2 Fig — (A) Line chart for early-warning signals in all symptomatic adults. (B) Line chart for early-warning signals in all asymptomatic adults. (C) Table of sDNB diagnoses and clinical diagnoses for all adults and samples. (PDF) [file pcbi.1005633.s002.pdf]
