# Supplementary material for: Quantifying critical states of complex diseases using single-sample dynamic network biomarkers
Source: PLoS Comput Biol. 2017 Jul 5;13(7):e1005633. doi: 10.1371/journal.pcbi.1005633 (PMC5517040; doi:10.1371/journal.pcbi.1005633)
Supplement: S13 Table — (XLSX) [file pcbi.1005633.s015.xlsx]

Table S13: the functional enrichment of the 54 overlapped genes among sDNB with p value of *sPCC* 0.05 and score of sDNB 1.6

| **Gene Ontology Consortium** | | **g:Profiler** | | **IPA** | |
| --- | --- | --- | --- | --- | --- |
| **enriched items** | **enriched *p* value** | **enriched items** | **enriched *p* value** | **enriched items** | **enriched *p* value** |
| defense response to virus (GO:0051607) | 4.55×10^-21^ | defense response to virus (GO:0051607) | 4.82×10^-17^ | Viral infection | 8.54×10^-17^ |
| response to virus (GO:0009615) | 1.45×10^-19^ | response to virus (GO:009615) | 1.23×10^-16^ | antiviral response | 1.63×10^-13^ |
| negative regulation of viral genome replication (GO:0045071) | 4.21×10^-09^ | immune response (GO:0006955) | 9.16×10^-16^ | antimicrobial response | 2.04×10^-13^ |
| negative regulation of viral process (GO:0048525) | 1.36×10^-08^ | defense response (GO:0006952) | 8.62×10^-15^ | replication of virus | 2.17×10^-12^ |
| negative regulation of viral life cycle (GO:1903901) | 4.83×10^-07^ | response to type I interferon (GO:0034340) | 1.6×10^-14^ | replication of RNA virus | 6.5×10^-11^ |
