# Supplementary material for: Quantifying critical states of complex diseases using single-sample dynamic network biomarkers
Source: PLoS Comput Biol. 2017 Jul 5;13(7):e1005633. doi: 10.1371/journal.pcbi.1005633 (PMC5517040; doi:10.1371/journal.pcbi.1005633)
Supplement: S1 Text — (DOC) [file pcbi.1005633.s016.doc]

**S1 Text. Deriving a criterion of single-sample dynamic network biomarkers**

Based on the DNB theory, there is a critical state during disease progression, which satisfies the following three conditions (see Introduction):

[Condition 1] deviation for molecules inside the module (*SD_in_*: standard deviation) drastically increases,

[Condition 2] correlation between molecules inside the module (*PCC_in_*: Pearson correlation coefficient in absolute value) rapidly increases, and

[Condition 3] correlation between molecules inside and outside the modules (*PCC_out_*: Pearson correlation coefficient in absolute value) rapidly decreases.

The three conditions of DNB are represented by the three terms in Eqn. (1), i.e., *SD_in_*, *PCC_in_*, and *PCC_out_*, when there are multiple samples. However, when there is only one sample, we approximately represent the three conditions in the following way, as shown in Eqn. (6).

1. For Condition 1

With respect to all reference samples (from the sample-1 to sample-n) and one additional sample (i.e. test sample-(n+1)), the standard deviation *SD_n+1_(x)* for all n+1 samples is

$\begin{matrix} SD_{n+1}(x)=\sqrt{\frac{\sum_{i=1}^{n+1} (x_{i}-\overline{x})^{2}}{n+1}}=\sqrt{\frac{\sum_{i=1}^{n} (x_{i}-\overline{x})^{2}}{n}}+\frac{1}{2n}(\frac{(x_{n+1}-\overline{x})^{2}}{\sqrt{\frac{\sum_{i=1}^{n} (x_{i}-\overline{x})^{2}}{n}}}-\sqrt{\frac{\sum_{i=1}^{n} (x_{i}-\overline{x})^{2}}{n}})+O(\frac{1}{n^{2}}) \\ =SD_{n}(x)+\frac{1}{2n}(\frac{(x_{n+1}-\overline{x})^{2}}{SD_{n}(x)}-SD_{n}(x))+O(\frac{1}{n^{2}}) \end{matrix}$,

where *O(1/n^2^)* is the term with the order equal to or higher than *1/n^2^,* and *SD_n_* is the standard deviation of *x* with n samples (the reference samples). With respect to all reference samples, one additional sample (i.e. test sample-(n+1)) makes the change of the standard deviation for variable *x* as

$SD_{n+1}(x)-SD_{n}(x)=\frac{1}{2n}(\frac{(x_{n+1}-\overline{x})^{2}}{SD_{n}(x)}-SD_{n}(x))+O(\frac{1}{n^{2}})$.

Clearly, the additional sample (sample-(n+1)) mainly affects the first term of the above change. Thus, we adopt the following expression to approximate the standard deviation of *x* for sample-(n+1):

$sED(x)=sSD(x)\approx\sqrt{(x_{n+1}-\overline{x})^{2}}=\left|x_{n+1}-\overline{x}\right|$,

which can be calculated in an efficient way. Here, *sED(x)* is the expression deviation of gene *x*, and further *sED_in_* indicates the average expression deviation *sED(x)* of all genes in the module for the sample-(n+1). Note that when n is sufficiently large, *SD_n+1_=SD_n_*+*O(1/n).*

1. For Condition 2

In the same way as the deviation, with respect to all reference samples (from sample-1 to sample-n), one additional sample (i.e. test sample-(n+1)) makes the change of the Pearson correlation coefficient inside the module as

$\begin{matrix} sPCC(x,y)=|PCC_{n+1}(x,y)-PCC_{n}(x,y)| \\ =|\frac{\sum_{i=1}^{n+1} (x_{i}-\overline{x})(y_{i}-\overline{y})}{SD_{n+1}(x)SD_{n+1}(y)}-\frac{\sum_{i=1}^{n} (x_{i}-\overline{x})(y_{i}-\overline{y})}{SD_{n}(x)SD_{n}(y)}| \\ =|PCC_{n}(x,y)(\frac{SD_{n}(x)SD_{n}(y)}{SD_{n+1}(x)SD_{n+1}(y)}-1)+\frac{(x_{n+1}-\overline{x})}{SD_{n}(x)}\frac{(y_{n+1}-\overline{y})}{SD_{n}(y)}\frac{SD_{n}(x)SD_{n}(y)}{SD_{n+1}(x)SD_{n+1}(y)}| \end{matrix}$ ,

where *sPCC(x,y)* is the differential PCC of *x* and *y* between *n+1* samples and *n* samples (the reference samples) in absolute value, and *sPCC_in_* is the average differential PCC between all *x* and y inside the module in absolute value. Clearly, when *n* is sufficiently large, *SD_n+1_=SD_n_*+*O(1/n)* (see the derivation in the above Condition 1) and thus we have

$\begin{matrix} sPCC(x,y){}={}|PCC_{n+1}(x,y)-PCC_{n}(x,y)|{}={}|\frac{(x_{n+1}-\overline{x})}{SD_{n}(x)}\frac{(y_{n+1}-\overline{y})}{SD_{n}(y)}|+O(\frac{1}{n}) \\ \approx|\frac{(x_{n+1}-\overline{x})}{SD_{n}(x)}\frac{(y_{n+1}-\overline{y})}{SD_{n}(y)}| \end{matrix}$ ,

which can be considered to be proportional to *PCC(x,y)* of the sample-(n+1) between all *x* and y inside the module. Actually, we can also directly use the above expression to approximately estimate *PCC(x,y)* of the sample-(n+1) in Eqn.(6).

1. For Condition 3

Similar to Condition 2, with respect to all reference samples (from sample-1 to sample-n), one additional sample (i.e. test sample-(n+1)) makes the change of the Pearson correlation coefficient between variables inside and outside the module as *sPCC(x,y),* and then *sPCC_out_* is the average differential PCC between all *x* inside the module and y outside the module in absolute value. When *n* is sufficiently large, *sPCC(x,y)* can be considered to be proportional to *PCC(x,y)* of the sample-(n+1) between all *x* inside the module and y outside the module.

Based on the three conditions, when the system, i.e., the sample-(n+1), approaches a critical point, both *SD_in_* and *PCC_in_* become big but *PCC_out_* becomes small.

Thus, the three conditions can be integrated as Eqn. (6) for the sample-(n+1), i.e.,

$I_{s}=\frac{sED_{in}\cdot sPCC_{in}}{sPCC_{out}}$ .
